# Comorbidities in Privately Insured South Africans With Systemic Lupus Erythematosus

**DOI:** 10.7759/cureus.55470

**Published:** 2024-03-04

**Authors:** Mbombo Henriette Ngandu Ntumba, Kavita Makan, Eustasius Musenge, Mohammed Tikly

**Affiliations:** 1 Department of Internal Medicine, School of Clinical Medicine, Chris Hani Baragwanath Academic Hospital, University of the Witwatersrand, Johannesburg, Johannesburg, ZAF; 2 Division of Rheumatology, Department of Internal Medicine, School of Clinical Medicine, Chris Hani Baragwanath Academic Hospital, University of the Witwatersrand, Johannesburg, Johannesburg, ZAF; 3 School of Public Health, University of the Witwatersrand, Johannesburg, Johannesburg, ZAF

**Keywords:** africa, hypertension, private sector, infections, comorbidities, systemic lupus erythematosus

## Abstract

Introduction

Comorbidities in systemic lupus erythematosus (SLE) impact negatively on health-related quality of life (HRQoL) and life expectancy. We investigated the frequency and spectrum of comorbidities in privately insured South Africans with SLE.

Methods

The data of SLE patients based on International Classification of Diseases, Tenth Revision (ICD-10) codes and insured with Discovery Health Medical Scheme (DHMS), South Africa, aged ≥16 years at diagnosis and with ≥6 months of follow-up were reviewed. Demographics, comorbidities listed in the Charlson comorbidity index (CCI), other common comorbidities, intercurrent illnesses, and drug therapy were documented.

Results

Of the 520 patients coded as SLE, 207 met the inclusion criteria. Most were females (90.8%), with a median (interquartile range {IQR}) age and follow-up duration of 39 (30.3-53.0) and 6.1 (3.7-8.1) years, respectively. All patients had at least one comorbidity; the most frequent CCI comorbidities were pulmonary disease (30.9%), congestive heart failure (CHF, 15%), and renal disease (14.0%). Other common comorbidities were hypertension (53.1%) and mood and anxiety disorders (46.9%). Urinary tract infections (UTIs, 37.7%) and pneumonia (33.8%) were common intercurrent illnesses. The independent predictors of CHF were renal disease (odds ratio {OR}=855), dyslipidemia (OR=15.3), and male gender (OR=43.0); the independent predictors of hypertension were age at diagnosis (OR=1.03), type 2 diabetes (OR=4.45), and renal disease (OR=4.34); and the independent predictors of mood and anxiety disorders were female gender (OR=3.98), stroke (OR=3.18), UTI (OR=2.39), and chloroquine use (OR=1.94).

Conclusion

In this study of privately insured South Africans with SLE, comorbidities were common, and all patients had at least one comorbidity. Hypertension, infections, and mood and anxiety disorders were the leading comorbidities overall, and pulmonary disease was the most common CCI comorbidity. There is an obvious need to formally study the burden of mental health disorders in South African SLE patients.

## Introduction

Systemic lupus erythematosus (SLE) is a chronic systemic autoimmune rheumatic disease characterized by multiorgan involvement. With advances in treatment, patients with SLE are living longer but at the risk of increased comorbidities. These comorbidities are related to both the disease and drugs used in the treatment of SLE [1]. Comorbidities impact not only health-related quality of life (HRQoL) but also life expectancy in SLE [2,3]. This has led to greater emphasis and awareness of the importance of regular screening and proactive prevention of comorbidities in SLE [4].

The spectrum of comorbidities in SLE varies by disease duration, age, ethnicity, socioeconomic conditions, and access to healthcare. Patients with younger onset of SLE are at the greatest risk of developing comorbidities related to the long-term use of corticosteroids and immunosuppressive drugs. Cardiovascular disease (CVD), stroke, and osteoporosis are more likely to occur in older patients and in those with a longer disease duration [5]. Ethnogeographic variations in comorbidities have been observed in several studies. The common comorbidities in SLE patients in the Western industrialized world are CVD, malignancies, renal disease, and infections [6]. German and UK studies have reported depression to be a common comorbidity [6,7], and thyroid disease is often seen in patients with SLE in Crete [7]. African Americans with SLE are more likely to have CVD, strokes, and diabetes when compared to Caucasian Americans [8]. Hypertension and tuberculosis (TB) are especially common in SLE patients in developing countries in Asia and Africa [9-11].

South Africa has a two-tier healthcare system where 84% of the indigent population is serviced by state-funded public healthcare facilities [12]. The remaining 16% of the population accesses private healthcare funded mainly through private medical insurance schemes. Previous studies on comorbidities in rheumatoid arthritis (RA) in South Africa showed that HIV and TB are common in the public sector [13] but less common in the private sector where hypothyroidism was common [14]. In the case of SLE, we previously found that deaths of SLE patients in the public sector were mostly from infections and chronic kidney disease (CKD) [15]. In a more recent study of comorbidities in SLE at the same public sector health facility, we found hypertension and infections to be the most common comorbidities [11].

In the absence of any published data on comorbidities in privately insured South Africans with SLE, we investigated the spectrum and burden of comorbidities listed in the Charlson comorbidity index (CCI) [16] and other common SLE-associated comorbidities in patients privately insured with Discovery Health Medical Scheme (DHMS), the largest open medical aid scheme in South Africa [17].

## Materials and methods

Anonymized data were obtained from DHMS (registration number: 1125) with the support and assistance of Discovery Health (Pty) Ltd, an accredited administrator and managed care provider for medical schemes. The inclusion criteria for the study were patients 1) with International Classification of Diseases, Tenth Revision (ICD-10) codes M32.9 (SLE unspecified), M32.8 (other forms of SLE), M32.11 (endocarditis in SLE), M32.12 (pericarditis in SLE), M32.14 (glomerular disease in SLE), and M32.19 (SLE with organ or system involvement); 2) aged ≥16 years at diagnosis; and 3) with ≥6 months of follow-up data anytime from January 2008 to December 2017. Excluded from the study were patients with overlap and other connective tissue diseases (CTD), M32.0 (drug-induced SLE) and L93 (discoid lupus). The study was approved by the Human Research Ethics Committee of the University of the Witwatersrand (approval number: M191128) and by DHMS. 

Comorbidities included in CCI comorbidities, other comorbidities of TB, hypertension, dyslipidemia, osteoporosis, osteonecrosis, mood disorders and anxiety disorders, and intercurrent illnesses of pneumonia, meningitis, and urinary tract infections (UTIs) were identified by appropriate ICD-10 codes (Table 1) [18].

**Table 1:** ICD-10 codes for comorbidities included in the systemic lupus erythematosus study of South Africans in the private sector ICD-10: International Classification of Diseases, Tenth Revision

Comorbidity	ICD-10 codes
Charlson comorbidities	
Acute myocardial infarction	I21, I22, and I252
Congestive heart failure	I50
Peripheral vascular disease	I71, I790, I739, R02, Z958, and Z959
Cerebral vascular accident	I60, I61, I62, I63, I65, I66, G450, G451, G452, G458, G459, G46, I64, G454, I670, I671, I672, I674, I675, I676, I677, I678, I679, I681, I682, I688, and I69
Dementia	F00, F01, F02, and F051
Pulmonary disease	J40, J41, J42, J44, J43, J45, J46, J47, J67, J44, J60, J61, J62, J63, J66, J64, and J65
Peptic ulcer	K25, K26, K27, and K28
Liver disease	K702, K703, K73, K717, K740, K742, K746, K743, K744, and K745
Diabetes	E109, E119, E139, E149, E101, E111, E133, E143, E104, E114, E135, and E145
Diabetes complications	E102, E112, E132, E142, E103, E113, E133, E143, E104, E114, E134, and E144
Paraplegia	G81, G041, G820, G821, and G822
Renal disease	N03, N052, N054, N055, N056, N072, N073, N074, N01, N18, N19, and N25
Cancer	C0, C1, C2, C3, C40, C41, C43, C45, C46, C47, C48, C49, C5, C6, C70, C71, C72, C73, C74, C75, C76, C80, C81, C82, C83, C84, C85, C883, C887, C889, C900, C901, C91, C92, C93, C940, C941, C942, C943, C9451, C947, C95, and C96
Cancers, metastatic	C77, C78, C79, and C80
Liver disease	K721,K729, K766, and K767
HIV	B20, B21, B22, and B24
Other comorbidities	
Hypertension	I10
Osteonecrosis	M87.9 and M87.1
Osteoporosis	M80 and M81
Dyslipidemia	78.5
Mood and anxiety disorders	F30-39 and F40-48
Infections	
Cutaneous	L08.8
Meningitis	G03.9
Pneumonia	J09, J10, J11, J12, J13, J14, J15, J16, J17, and J18
Urinary tract	N39, N30, N30.00, N30.01, and N34
Tuberculosis	A15.0, A15.1, A15.2, A15.4, A15.7, A17.8, A18.0 A18.1, A18.3, A18.4, A18.5, A18.6, A18.7, and M49.0

The South African National Pharmaceutical Product Index (NAPPI) codes were used to document the following drugs: chloroquine, oral prednisone, cyclophosphamide, azathioprine, and methotrexate (Table 2). No biologic therapies were captured.

**Table 2:** National Pharmaceutical Product Index (NAPPI) codes for drugs included in the systemic lupus erythematosus study of South Africans in the private sector

Drug	NAPPI codes
Azathioprine	701252, 706108, 712609, and 700777
Chloroquine	747297 and 794333
Cyclophosphamide	723274
Prednisone	788783, 752304, and 818267
Methotrexate	712504 and 742465

Statistical analysis

Continuous variables were expressed as mean (SD) and median (interquartile range {IQR}) for normal and non-normal distributed data, respectively. The Mann-Whitney test was used to compare continuous variables between groups and chi-square/Fisher’s exact two-tailed tests for categorical variables. Univariate and multivariate logistic regression models were used to determine the independent predictors of common comorbidities. Variables with a P value <0.15 on univariate analysis were included in the multivariate analysis using the enter method of logistic regression analysis. A P value of <0.05 was deemed to be significant. All statistical analyses were performed using MedCalc version 20.009 for Windows (MedCalc Software Ltd, Ostend, Belgium).

## Results

Of the 520 patients with the relevant ICD-10 SLE codes identified on the DHMS database, 207 met the other inclusion/exclusion criteria. Most were females (90.8%), with a median (IQR) age and follow-up duration of 39.0 (30-53) and 6.1 (3.7-8.1) years, respectively (Table 3).

**Table 3:** Demographics and comorbidities in 207 South African privately insured patients with systemic lupus erythematosus OR, odds ratio; CI, confidence interval; IQR, interquartile range; NS, not significant

Variable	All (n=207)	Females (n=188)	Males (n=19)	P value
Age, median (IQR) in years	39 (30.3-53.0)	39 (31.0-52.5)	38 (29.3-61.0)	NS
Follow-up, median (IQR) in years	6.1 (3.7-8.1)	6.1 (3.7-8.1)	7.1 (3.7-8.1)	NS
Comorbidities of Charlson comorbidity index				
Acute myocardial infarction	3 (1.4)	2 (1.1)	1 (5.3)	NS
Congestive heart failure	31 (15.0)	23 (12.2)	8 (42.1)	0.002, OR (95% CI)=5.2 (2-14)
Peripheral vascular disease	0 (0)	0 (0)	0 (0)	NS
Cerebrovascular accident	27 (13.0)	27 (14.4)	0 (0)	NS
Paraplegia	2 (1.0)	1 (0.5)	1 (5.3)	NS
Dementia	1 (0.5)	1 (0.5)	0 (0)	NS
Peptic ulcer disease	27 (13.0)	27 (14.4)	0 (0)	NS
Liver disease (all)	4 (1.9)	4 (2.1)	0 (0)	NS
Mild	1 (0.5)	1 (0.5)	0 (0)	NS
Severe	3 (1.4)	3 (1.6)	0 (0)	NS
Diabetes (all)	23 (12.2)	26 (12.6)	3 (15.8)	NS
Complicated	3 (1.4)	2 (1.1)	1 (5.3)	NS
Pulmonary disease	64 (30.9)	60 (31.9)	4 (21.1)	NS
Chronic kidney disease	30 (14.5)	24 (12.8)	6 (31.6)	0.04, OR (95% CI)=3.2 (1.09-9.08)
Tumors (all)	12 (5.8)	11 (5.9)	1 (5.3)	NS
Localized	10 (4.8)	9 (4.8)	1 (5.3)	NS
Metastatic	2 (1.0)	2 (1.1)	0 (0)	NS
HIV	4 (1.9)	3 (1.6)	1 (5.3)	NS
Other comorbidities				
Hypertension	110 (53.1)	97 (51.6)	13 (68.4)	NS
Dyslipidemia	42 (29.3)	39 (20.7)	3 (15.7)	NS
Osteoporosis	29 (14.0)	29 (15.4)	0 (0)	NS
Avascular necrosis	1 (0.5)	1 (0.5)	0 (0)	NS
Mood and anxiety disorders	97 (46.9)	94 (50.0)	3 (15.7)	0.007, OR (95% CI)=5.3 (1.5-19)
Tuberculosis	2 (1.0)	1 (0.5)	1 (5.3)	NS
Intercurrent illnesses				
Urinary tract infection	78 (37.7)	72 (38.3)	6 (31.6)	NS
Pneumonia	70 (33.8)	63 (33.5)	7 (36.8)	NS
Meningitis	3 (1.4)	3 (1.6)	0 (0)	NS

All patients had at least one comorbidity, and two-thirds had at least one CCI comorbidity of which the most were pulmonary disease (30.9%), with bronchitis (45%) and asthma (36%) being especially common; congestive heart failure (CHF) (15%); and CKD (14.0%) (Table 2). Other common comorbidities and intercurrent illnesses were hypertension (53.1%); mood and anxiety disorders (46.9%); infections, particularly UTI (37.7%); and pneumonia (33.8%). Oral prednisone had been prescribed in 80 (38.6%), chloroquine in 131 (63.2%), and the immunosuppressive drugs, methotrexate, cyclophosphamide, and azathioprine in 44 (21%), four (1.9%), and 23 (11.1%) patients, respectively.

The independent associations/predictors of the most common comorbidities are shown in Table 4.

**Table 4:** Independent associations and predictors of major comorbidities in 207 South African patients with systemic lupus erythematosus OR, odds ratio; CI, confidence interval

Outcome variable	OR (95% CI)	P value
Congestive heart failure		
Male gender	43.0 (2.4-776.2)	0.01
Chronic kidney disease	855 (69-10668)	<0.0001
Dyslipidemia	15.3 (1.4-171.5)	0.02
Type 2 diabetes		
Duration of follow-up	1.21 (1.00-1.46)	0.004
Dyslipidemia	3.57 (1.35-9.48)	0.01
Prednisone therapy	2.47 (1.00-6.07)	0.05
Hypertension		
Age at diagnosis	1.03 (1.00-1.05)	0.02
Type 2 diabetes	4.45 (1.41-14.06)	0.01
Chronic kidney disease	4.34 (1.62-11.62)	0.004
Pulmonary disease		
Pneumonia	2.99 (1.56-5.7)	0.001
Dyslipidemia	2.59 (1.15-5.89)	0.02
Congestive heart failure	0.2 (0.06-0.64)	0.01
Peptic ulcer disease		
Pneumonia	5.49 (2.13-14.18)	0.0004
Type 2 diabetes	4.01 (1.32-12.19)	0.01
Urinary tract infections		
Pneumonia	1.95 (1.04-3.7)	0.04
Mood and anxiety disorders	2.18 (1.19-3.99)	0.01
Pneumonia		
Peptic ulcer disease	2.80 (1.01-7.80)	0.05
Pulmonary disease	2.31 (1.13-4.69)	0.02
Urinary tract infections	2.17 (1.09-4.3)	0.03
Mood and anxiety disorders		
Female gender	3.98 (1.07-14.79)	0.04
Cerebrovascular accident	3.18 (1.2-842)	0.02
Urinary tract infection	2.39 (1.27-4.48)	0.007
Chloroquine therapy	1.94 (1.03-3.65)	0.04

Chronic kidney disease was the strongest predictor of CHF. Apart from age and dyslipidemia, prednisone use was an independent predictor of type 2 diabetes. Both hypertension and CKD were strongly associated with type 2 diabetes. There was a significant association of pulmonary disease with pneumonia; the latter is also associated with peptic ulcer disease. Mood and anxiety disorders were more common in females, those with a stroke, and those with chloroquine use and a bidirectional association of mood and anxiety disorders with UTIs.

## Discussion

In this study of privately insured South Africans with SLE, all patients had at least one comorbidity as compared to a study from the United Kingdom in whom about 50% of the patients had no documented CCI comorbidity [5], and in our earlier study of public sector SLE patients, 36% of the patients had at least one comorbidity at diagnosis, which increased to 56% after a median follow-up period of seven years [11]. The differences in the frequency, spectrum, and prevalence of comorbidities can be explained by true differences in the prevalence of comorbidities in the background general population and variations in study design and the documentation of comorbidities. For example, in the case of the UK study, Read codes, which are a coded thesaurus of clinical terms of the National Health Service, were used [5], whereas comorbidity data were extracted from clinical case records in our study of public sector patients in South Africa [11].

Moreover, the spectrum of comorbidities in this privately insured cohort differs from the public sector: hypertension and mood and anxiety disorders were especially prevalent in this cohort, whereas in the public sector, serious infections and TB were especially common [11,19]. A meta-analysis of SLE studies in Africa showed that infections are common and the prevalence of hypertension and CHF is 10.3%-19.6% and 33.3%, respectively [20].

Pulmonary comorbidities, particularly chronic obstructive pulmonary disease (COPD) and asthma, have been reported previously to occur in 9.3%-19.3% of SLE patients [6,7,21] compared to 30% in the current study, of which a third was asthma. SLE patients have a higher risk of asthma than the general population [22], and asthma negatively impacts HRQoL [21]. Smoking has been proposed as the common link between SLE and COPD since it is known to be a trigger for SLE and a risk factor for COPD [23].

Mood and anxiety disorders have been increasingly recognized in SLE. Some studies have reported mental health disorders in up to 40% of SLE patients [7], and the prevalence of depression ranges globally from 20% to 30% [6,24]. A qualitative South African study on various aspects of living with SLE showed that a fifth of the patients needed a referral for the management of depression [25]. Half the patients in the current study were documented to have a mood and/or anxiety disorder, especially in females, those who had a stroke or UTI, and those treated with chloroquine. These findings are consistent with those of Bai et al. where urinary symptoms, proteinuria, overall disease activity, and the cumulative dose of hydroxychloroquine were associated with depression [26].

Like our findings of SLE in the public sector, CVD was common, but coronary artery disease (CAD), a leading cause of death in SLE in Western populations, was rare despite the high prevalence of traditional CAD risk factors [11]. It is plausible that at least some patients with CHF had underlying CAD given that the independent risk factors for CHF, CKD, dyslipidemia, and male gender, in the current study are all known risk factors for CAD. The prevalence of CHF in a US population-based SLE study was 2.5%, and myopericardial and valvular heart diseases were risk factors for early CHF, nephritis, and hypertension for delayed-onset heart failure and black race for both early- and delayed-onset CHF [27].

Lupus nephritis, often leading to CKD, is especially common in SLE patients of African extraction [15,20]. Not surprisingly, 14% of the patients had CKD in the current study, substantially higher than the 7.7%-9.5% reported in European studies [6,7]. Consistent with the findings in a multicenter Swedish study, CKD was more significantly common in males than females [28].

Infections are a major cause of morbidity and mortality in SLE globally [19,29]. The increased risk of infection in SLE has been attributed to immune dysregulation, functional asplenia, and drug therapy with corticosteroids and other immunosuppressive agents [30]. The relative risk of infections in SLE is increased two- to sixfold compared to the general population [29]. Unlike in previous South African studies where approximately one in six patients contracted TB [10], TB was rare in the current study. This is likely a reflection of socioeconomic disparities between patients cared for in the public and private sectors where TB is more common in the lower socioeconomic group [31]. As mentioned previously, the public sector services 84% of the population, yet health expenditure is roughly the same for both the private and public sectors, about US$55 billion each annually [12].

The present study has some limitations. Firstly, the diagnosis of SLE and comorbidities was based solely on ICD-10 codes without case record verification. Secondly, we were unable to determine the duration of disease, ethnicity, and smoking history, all of which are factors known to impact the burden and spectrum of comorbidities. Thirdly, the small sample size of 207 patients limits the statistical power of the study. Fourthly, it was not possible to explore the temporal relationship of comorbidities with intercurrent illnesses, e.g., UTI with mood disorders, to determine a possible causal relationship. Finally, we could not access mortality data to determine the relationship of comorbidities with mortality.

## Conclusions

In summary, our findings show that comorbidities are common in privately insured South Africans with SLE, but the spectrum differs from the public sector. COPD and mental health disorders were especially common. There were substantially less TB and HIV infections documented compared to the public sector. There is an obvious need to formally study the burden of mental health disorders in South African SLE patients. Health professionals, especially those working in the public sector, need to be more aware of the mental health needs of SLE patients.
